# Catalpol inhibits apoptosis in hydrogen peroxide-induced cardiac myocytes through a mitochondrial-dependent caspase pathway

**DOI:** 10.1042/BSR20160132

**Published:** 2016-06-30

**Authors:** Ling-ai Hu, Yu-kun Sun, Hong-sheng Zhang, Jin-guo Zhang, Jian Hu

**Affiliations:** * Department of Cardiology, The Affiliated Hospital of Jining Medical College, Jining, Shandong 272100, China; † Department of Prosthodontics, Jining Stomatology Hospital, Jining, Shandong 272100, China; ‡ Department of Cardiology, The First Affiliated Hospital of China Medical University, Shenyang, Liaoning 110001, China

**Keywords:** apoptosis, Catalpol, H9c2, hydrogen peroxide

## Abstract

Catalpol, an iridoid glucoside, has been reported to inhibit apoptosis of neuron and endothelial cells. In the present study, we investigated the mechanism of catalpol-mediated cardioprotection. The rat embryonic ventricular myocardial cell line (H9c2) cells were first incubated with catalpol, and then exposed to hydrogen peroxide (H_2_O_2_). The concentration of malondialdehyde (MDA) and the activity of superoxide dismutase (SOD) were all determined by using commercially available kits. Apoptotic cells were assessed by Hoechst 33258 and Annexin V-fluorescein isothiocyanate binding assay. Synthesis of Bcl-2, Bax, cytochrome *c* and caspase-3 were analysed by real-time semiquantitative reverse transcription-PCR and Western blotting. We observed that apoptosis in H9c2 was associated with increased Bax, cytochrome *c*, caspase-3, decreased Bcl-2 activity after 24 h of H_2_O_2_ exposure. Catalpol pretreatment afforded a marked protection against the above H_2_O_2_-mediated cytotoxicity and apoptosis in H9c2 cells. Moreover, the catalpol pretreatment led to a great reduction in H_2_O_2_-induced MDA release and increased SOD. These findings indicated for the first time that pretreatment of H9c2 cells with catalpol can be against H_2_O_2_-induced apoptosis, and the protective effect of catalpol involves the mitochondrial-dependent caspase pathway and is associated with increased Bcl-2 and decreased Bax expression.

## Abstract (precis)

These findings indicated for the first time that pretreatment of H9c2 cells with catalpol can against H_2_O_2_-induced apoptosis, and the protective effect of catalpol via a mitochondrial-dependent caspase pathway and is associated with increased Bcl-2 and decreased Bax expression.

## INTRODUCTION

Apoptosis or programmed cell death plays a key role in the maintenance of homoeostasis of both normal development and many pathologic conditions. Oxidative stress is a well-known factor that not only promoted apoptosis [1], but also implicated in the pathophysiology of a number of cardiovascular disorders such as ischaemia-reperfusion injury [2], atherosclerosis [3], chronic heart failure [4], hypertrophy [5] and hypertension [6]. Therefore, modification of apoptotic pathways to attenuate cardiomyocyte damage induced by myocardial oxidative stress is a major area of clinical interest.

Catalpol, the first iridoid glycoside isolated and identified from the radix of *Rehmannia glutinosa* Libosch, has been shown to have many important and extensive pharmacological action, including hypoglycaemic [7], diuretic [8], anti-cancer [9], anti-spasmodic, anti-inflammatory [10] and anti-apoptotic [11], based on *in vitro* and *in vivo* pharmacodynamic experiments. Catalpol has also been proved to promote cell survival in a number of cell types such as human umbilical vein endothelial cells (HUVECs) by suppressing the production of free radicals and elevating antioxidant capacity in recent years [11]. However, the effects of catalpol on the oxidative stress have not been elucidated in cardiac muscle cells.

The Bcl-2 family proteins are central regulators for cell apoptosis under both normal and oxidative conditions [12]. Mitochondrial disruption and the subsequent release of pro-apoptotic proteins like cytochrome *c* is a critical event, which impinges on the process of apoptosis involving multiple cellular events [13]. The release of these factors from the mitochondria is regulated by Bcl-2 family proteins, which are divided into two subgroups: pro-survival, e.g. Bcl-2, Bcl-XL, and pro-death, e.g. Bax, Bak [14–16]. Post-translational modification and/or increases in the expression of proapoptotic genes are another way to initiate apoptosis [13,17,18].

In the present study, we examined the effect of catalpol on H_2_O_2_-induced apoptosis in the rat embryonic ventricular myocardial cell line (H9c2). We also examined potential mechanisms underlying catalpol-associated protection, including alterations in the mitochondrial-dependent caspase pathway and changes in Bcl-2 and Bax expression.

## MATERIALS AND METHODS

### Cell culture

H9c2 cells were obtained from A.T.C.C. and maintained in 100 ml culture flask and maintained by growth medium, which consists of Dulbecco's modified Eagle's medium (DMEM), 10% heat-inactivated FBS (TBD), 100 unit/ml penicillin and 100 mg/ml streptomycin. Cells were incubated in a humidified incubator containing 5% CO_2_ at 37°C and subcultured when they reached 80–90% confluence.

### Experimental protocol

There were five groups in the present study: (1) cells were untreated (control group); (2) cells exposed to 100 μM H_2_O_2_ for 24 h (H_2_O_2_ group); and (3–5) cells coincubated with catalpol and H_2_O_2_ (catalpol group). Cells were exposed to catalpol (0.1–10 μg/ml) 24 h prior to, as well during the H_2_O_2_ exposure period (unless indicated otherwise). This concentration was chosen based on our preliminary experiments.

### Assessment of cell damage

Cellular damage was determined by colorimetric MTT assay (Sigma) following the manufacturer's protocols. After incubation of the cells with each treatment, MTT was added to each well to a final concentration of 0.5 mg/ml for 4 h at 37°C. The supernatants were removed and 150 μl of DMSO was added to each well to dissolve the formazan crystals at room temperature. Absorbance was measured at 490 nm using an ELISA reader (Sunrise RC). Cell viability was expressed as a percentage of the control culture.

Lactate dehydrogenase (LDH) leakage was determined using a LDH-cytotoxic test kit (Jiancheng Bioengineering). Briefly, at the end of the treatment period, 20 μl of culture medium was added to the reaction mixture containing NAD+ and 2 mM pyruvate for 15 min at 37°C. Then colouring reagent was added and incubated for a further 15 min, and stopped by the addition of 0.4 M NaOH. Measure the absorbance at 440 nm using an ELISA microplate reader (Sunrise RC).

### Measurement of cellular superoxide dismutase content, malondialdehyde levels

The supernatants separated were used for measurement of cellular superoxide dismutase (SOD) content, malondialdehyde (MDA) levels using the commercially available colorimetric assay kits respectively (Jiancheng Bioengineering). The content of SOD can be assayed by its ability to inhibit the oxidation of hydroxylamine in the xanthine–xanthine oxidase system. One unit of SOD activity was defined as the amount that reduced the absorbance at 550 nm by 50%. The concentration of MDA can be measured at a wavelength of 532 nm on the basis of its reaction with thiobarbituric acid to form a pink-coloured complex. The level of MDA was expressed as nmol MDA per milligram protein.

### Morphological assay

H9c2 cells (1×10^5^ cells/ml) were treated with catalpol for 24 h and then replaced in DMEM with H_2_O_2_ for 24 h at 37°C, cells were fixed with 4% paraformaldehyde for 20 min at room temperature and incubated with 10 μg/ml Hoechst 33258 for 10 min at 37°C, after washing three times, Hoechst-stained cells were viewed and photographed under a fluorescence microscope (Tokyo, Olympus).

### Annexin V-FITC method

After the incubation period, cells were trypsinized into a single cell suspension, and resuspended in 100 μl of binding buffer containing 10 mM Hepes/NaOH pH 7.4, 140 mM NaCl and 2.5 mM CaCl_2_. Five microlitres Annexin V-FITC and 5 μl of propidium iodide (PI, 20 μg/ml: Jingmei Biotechnology) were labelled with the cells in the dark for 15 min. After incubation, the cells were resuspended in 400 μl of binding buffer and then apoptosis analysis was performed by flow cytometry (FACScalibur, Becton-Dickinson). The percentages of apoptotic cells were calculated by counting the Annexin V-positive cells relative to the total cell population.

### Western blotting analysis

Cells were scraped into lysis buffer (50 mM Tris/HCl, 150 mM NaCl, 1% Nonidet P-40, 0.5% sodium deoxycholate, 0.1% SDS (pH 7.5) and 1 mM PMSF) and the protein content of the supernatant was assayed with folin method (UV300). Each supernatant containing 100 μg of protein except that used for cytochrome *c* was boiled for 5 min in sample buffer (50 mM Tris/HCl pH 6.8, 12.5% glycerol, 1% SDS, 0.01% Bromophenol Blue) containing 5% β-mercaptoethanol. The proteins were loaded on a (12%) SDS/PAGE and followed by electrophoretically transfer to a PVDF membrane. Nonspecific binding sites were blocked with 5% non-fat dried milk overnight, rinsed with TBS two times and then incubated with primary antibodies for Bcl-2, Bax, cytochrome *c* or caspase-3 (1:400, Boster) at 4°C overnight. Blots were then washed with TBS two times and treated with the respective alkaline phosphatase-tagged secondary antibody (1:10000, Boster). Bands were detected using chromogenic substrate alkaline phosphatase and quantified with reference to the reference protein β-actin by densitometry.

### Semiquantitative real-time RT-PCR

Total RNA was extracted from cells using TRIZOL reagent following the manufacturer's instructions (Invitrogen, Life Technologies). RNA concentrations were quantified by absorbance measurements at 260 and 280 nm using a spectrophotometer (UV300). Reverse transcription was performed with Revert Aid H Minus M-muLV reverse transcriptase (Biometra). Real-time RT-PCR was carried out in a volume of 20 μl reaction mixture which contained 2 μl cDNA, 10 μl SYBR® Premix Ex Taq™ 0.4 μl of each primer (10 μM) and 7.2 μl ultrapure water using an ABI 7700 Prism Sequence Detection System (Applied Biosystems) according to the manufacturer's protocol and using the following primers: Bcl-2: Forward: 5′-TACGAGTGGGATACTGGAGA-3′3CGAGReverse: 5′-TCAGGCTGGAAGGAGAAG-3′; Bax: Forward: 5′-GTTACAGGGTTTCATCCAGG-3′ and Reverse: 5′-CGTGTCCACGTCAGCAAT-3′; cytochrome *c*: Forward: 5′-AAATGGGTGATGTTGAA-3′ and Reverse: 5′-TTGGTC-CAGTCTTATGC-3′; caspase-3: Forward: 5′-CTGGACTGCG-GTATTGAG-3′ and Reverse: 5′-GGGTGCGGTAGAGTAAGC-3′; β-actin: Forward: 5′-CGTGCGTGACATTAAAGAG-3′ and Reverse: 5′-TTGCCGATAGTGATGACCT-3′. The expression of β-actin was used as the internal standard.

### Statistical analysis

All data are presented as mean±S.D. Differences between mean values of multiple groups were analysed by one-way ANOVA. The results were considered to be statistically significant when *P*<0.05.

## RESULTS

### Effect of catalpol on the viability of H9c2 exposed to H_2_O_2_

**Figure 1:**
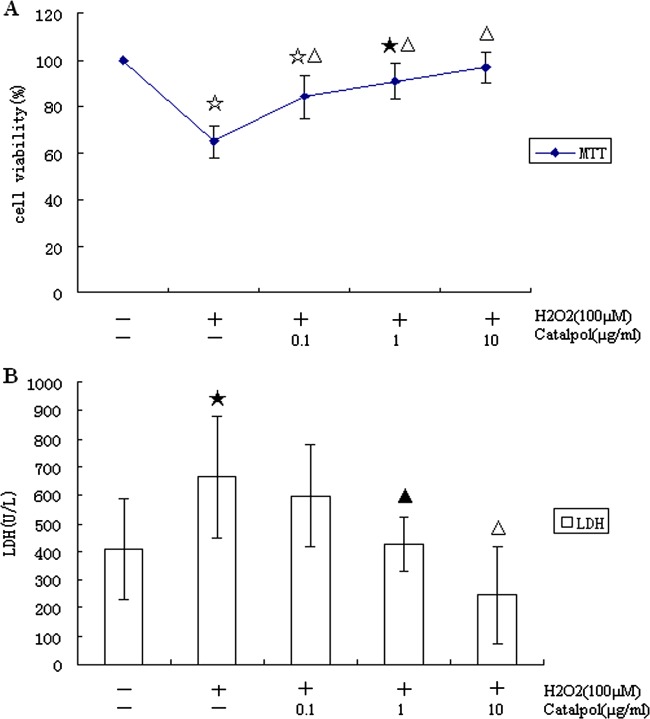
Protective effect of catalpol on viability losses in H9c2 induced by H_2_O_2_ (100 μM) H9c2 were preincubated with 0.1–10 μg/ml of catalpol for 24 h and then exposed to H_2_O_2_ for 24 h. (**A**) Preventive effects of catalpol on cell viability against H_2_O_2_-induced injury determined by MTT assay. (**B**) Inhibition of LDH release in H9c2 by catalpol after exposure to H_2_O_2_. Values are means±S.D. from five independent experiments. ^★^*P*<0.05 and ^☆^*P*<0.01 compared with control group, ^▲^*P*<0.05 and ^△^*P*<0.01 compared with H_2_O_2_ group.

The results in Figures 1(A) and 1(B) showed that incubation of H9c2 cells with 100 μM H_2_O_2_ resulted in a dramatic decline of cell viability and LDH levels, which decreased to 64.90±7.01% and 665.69±217.07 unit/l respectively. Preincubation of cells with 0.1, 1 or 10 μg/ml of catalpol resulted in a dose-dependent manner variation of cell viability and LDH content, which were increased up to 83.9±9.2%, 90.9±7.7%, 96.6±6.6% and 597.94±180.06 unit/l, 426.47±96.21 unit/l, 247.06±172.26 unit/l respectively.

### Effect of catalpol on cellular SOD content, MDA level in H9c2 exposed to H_2_O_2_

**Figure 2:**
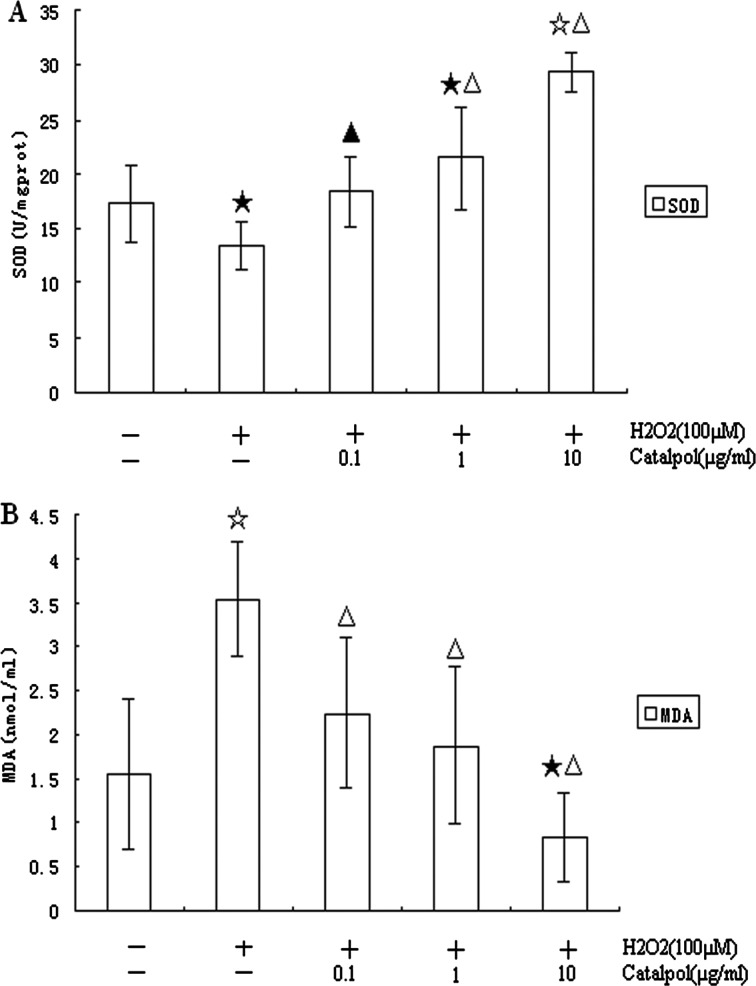
Protective effect of catalpol on SOD content and MDA level in H9c2 induced by H_2_O_2_ (100 μM) H9c2 were preincubated with 0.1–10 μg/ml of catalpol for 24 h and then exposed to H_2_O_2_ for 24 h. (**A**) Facilitation release of SOD in H9c2 by catalpol after exposure to H_2_O_2_. (**B**) Inhibition of MDA release in H9c2 by catalpol after exposure to H_2_O_2_. Values are means±S.D. from five independent experiments. ^★^*P*<0.05 and ^☆^*P*<0.01 compared with control group, ^▲^*P*<0.05 and ^△^*P*<0.01 compared with H_2_O_2_ group.

As shown in Figures 2(A) and 2(B), compared with the control (17.34±3.58 unit/mg · protein), treatment of H9c2 cells with 100 μM of H_2_O_2_ for 24 h caused significantly less activities of SOD (13.41±2.08 unit/mg · protein). However, catalpol (0.1, 1 or 10 μg/ml) pretreatment significantly increased the activities of SOD in a dose-dependent fashion (18.36±3.23, 21.48±4.67, 29.35±1.84 unit/mg · protein respectively) compared with the H_2_O_2_ group. In addition, H9c2 cells treated with 100 μM of H_2_O_2_ for 24 h caused more MDA levels (3.54±0.64 nmol/mg · protein), whereas preincubation of cells with catalpol (0.1, 1 or 10 μg/ml) markedly attenuated the increases (2.25±0.84, 1.88±0.88, 0.83±0.51 nmol/mg · protein respectively) compared with the H_2_O_2_ group.

### Effect of catalpol on apoptosis in H9c2 exposed to H_2_O_2_

**Figure 3:**
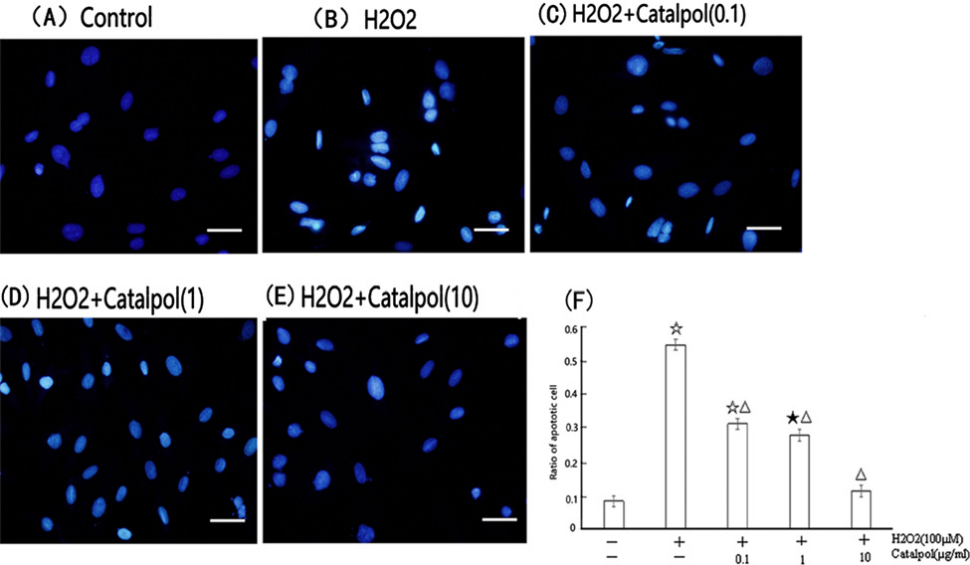
The morphological changes of H9c2 cells shown by Hoechst 33258 staining The cells were observed by fluorescence microscopy (400×) after nuclei staining with Hoechst 33258. (**A**) Control group; (**B**) H_2_O_2_ group; (**C**) 0.1 μg/ml catalpol group; (**D**) 1 μg/ml catalpol group; (**E**) 10 μg/ml catalpol group; (**F**) quantification results of the apoptotic nuclei.

Round-shaped nuclei with homogeneous fluorescence intensity is shown in the control group (Figure 3A). However, a marked increase in apoptotic cells which contained heterogeneous intensity, chromatin condensation and fragmentation appeared after 24 h treatment with 100 μM H_2_O_2_ (Figure 3B). Pretreatment of catalpol completely protected cells from morphological changes by H_2_O_2_ (Figures 3C and 3D).

**Figure 4:**
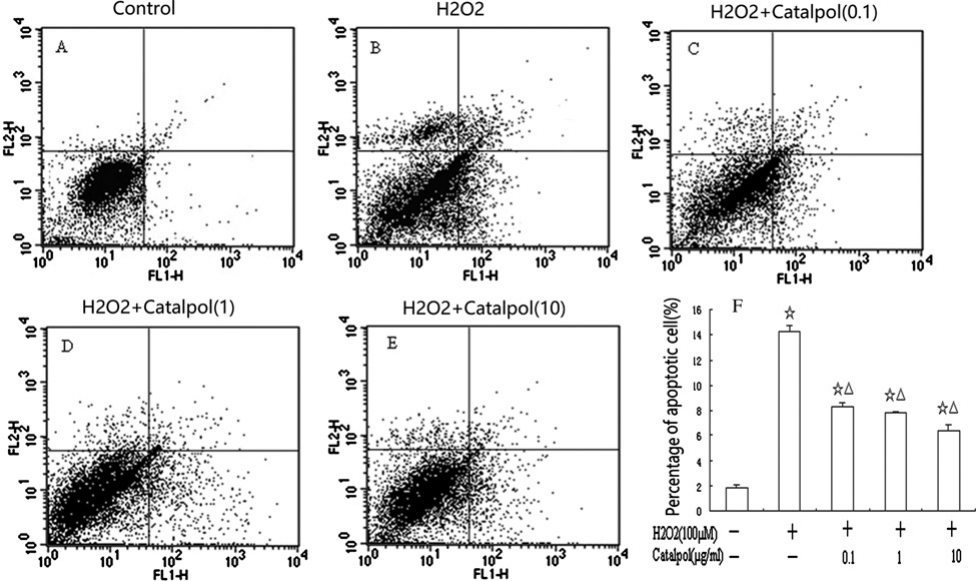
Flow-cytometric analysis of apoptosis in H9c2 cells that were pretreated with catalpol H9c2 were treated with 0.1–10 μg/ml of catalpol for 24 h and then with 100 μM of H_2_O_2_ for another 24 h at 37°C. After incubation, cells were harvested and labelled with a combination of Annexin V-FITC and PI. (**A**) Control group; (**B**) H_2_O_2_ group; (**C**) 0.1 μg/ml catalpol group; (**D**) 1 μg/ml catalpol group; (**E**) 10 μg/ml catalpol group; (**F**) shows a column bar graph analysis. Values represent means±S.D. from three independent experiments. ^☆^*P*<0.01 compared with control group, ^△^*P*<0.01 compared with H_2_O_2_ group.

Exposure of H9c2 cells to 100 μM H_2_O_2_ for 24 h resulted in an increase in cellular apoptosis (14.30±0.41%) as revealed by flow cytometry (Figures 4B and 4F). Pretreatment of cells with catalpol (0.1, 1 or 10 μg/ml) for 24 h prior to H_2_O_2_ reduced the percentage of apoptotic cells to 8.97±0.36, 7.81±0.06 and 6.38±0.43%, respectively, in a concentration-dependent manner (Figures 4C–4F).

### Effect of catalpol on the expression of Bcl-2 and Bax in H9c2 exposed to H_2_O_2_

**Figure 5:**
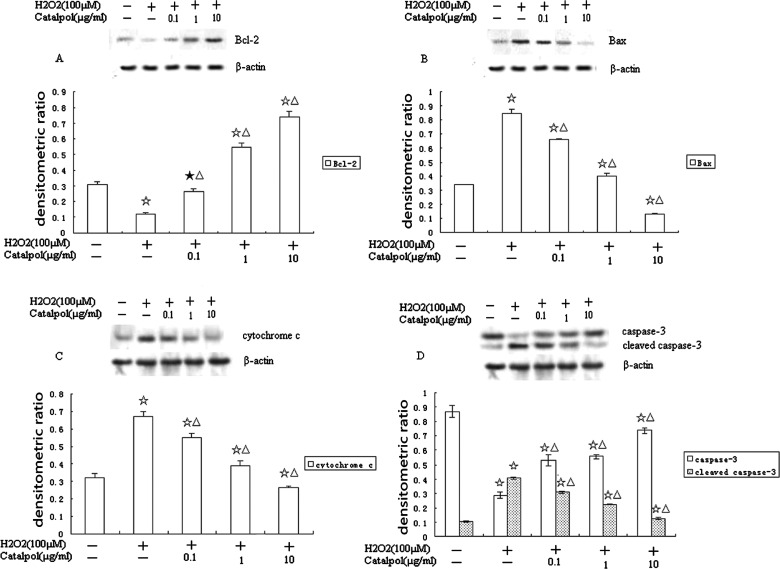
Representative Western blotting showing the protein expression (**A**) Bcl-2; (**B**) Bax; (**C**) cytochrome *c*; (**D**) caspase-3; Groups: control group; H_2_O_2_ group; 0.1 μg/ml catalpol group; 1 μg/ml catalpol group; and 10 μg/ml catalpol group. Data were expressed as densitometric ratio. Values are mean±S.D. (*n*=3). ^★^*P*<0.05 and ^☆^*P*<0.01 compared with control group, ^△^*P*<0.01 compared with H_2_O_2_ group.

We investigated the protein and mRNA expression of the anti-apoptotic Bcl-2 and the pro-apoptotic Bax analysed by Western blotting and real time RT-PCR. The results in Figures 5(A) and 5(B) showed, the level of Bax in H9c2 cells with H_2_O_2_ increased significantly to 0.85±0.03 and Bcl-2 decreased significantly to 0.12±0.01, compared with the control group (Bax: 0.34±0.01; Bcl-2: 0.31±0.02). Whereas preincubation with catalpol (0.1, 1 or 10 μg/ml) significantly reduced the level of Bax (0.66±0.01, 0.40±0.02, 0.13±0.01 respectively) and the level of Bcl-2 (0.26±0.02, 0.55±0.02, 0.74±0.04 respectively) induced by H_2_O_2_ in a concentration-dependent manner, compared with the H_2_O_2_ group.

**Figure 6:**
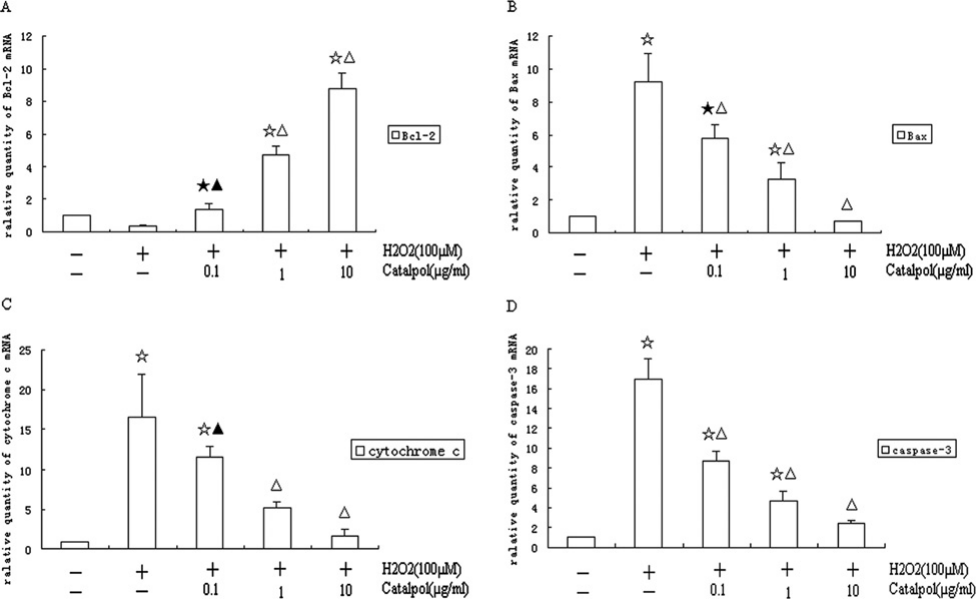
Bcl-2, Bax, cytochrome *c* and caspase-3 mRNA expression in H9c2 cells Cells were treated with catalpol (0.1–10 μg/ml) for 24 h, and then incubated with 100 μM of H_2_O_2_ for another 24 h at 37°C. After incubation, cells were harvested and used real-time RT-PCR analysis. (**A**) Bcl-2; (**B**) Bax; (**C**) cytochrome *c*; (**D**) caspase-3. Normalization relative to β-actin was performed. Results presented in bar graph are the means±S.D. from three independent experiments. ^★^*P*<0.05 and ^☆^*P*<0.01 compared with control group, ^▲^*P*<0.05 and ^△^*P*<0.01 compared with H_2_O_2_ group.

Incubation with H_2_O_2_ (100 μM) for 24 h significantly inhibited the mRNA expression of Bcl-2 (0.36-fold, Figure 6A) and increased the expression of Bax (9.15-fold, Figure 6B). Catalpol at 0.1, 1 or 10 μg/ml significantly increased the Bcl-2 mRNA level (1.35-fold, 4.69-fold, 8.79-fold, respectively, Figure 6A) and decreased the Bax mRNA level (5.75-fold, 3.27-fold, 0.74-fold, respectively, Figure 6B), compared with control group.

### Effect of catalpol on the activity of cytochrome *c* and caspase-3 in H9c2 exposed to H_2_O_2_

The protein levels of cytochrome *c* and caspase-3 were analysed by Western blotting. As shown in Figures 5(C) and 5(D), the activity of caspase-3 (cleaved caspase-3) and cytochrome *c* were significantly increased after H_2_O_2_ treatment (0.41±0.01, 0.67±0.03 respectively). However, catalpol at 0.1, 1 or 10 μg/ml abolished the activation of caspase-3 (0.34±0.02, 0.22±0.01, 0.13±0.01 respectively) and the release of cytochrome *c* (0.55±0.02, 0.39±0.03, 0.26±0.01 respectively) in H_2_O_2_-treated cells in a concentration-dependent manner.

The mRNA levels of cytochrome *c* and caspase-3 were analysed by real time RT-PCR. As shown in Figures 6(C) and 6(D), the addition of H_2_O_2_ to the cells increased the mRNA expression of cytochrome *c* (16.60-fold relative to control) and caspase-3 (16.96-fold relative to control). Catalpol pretreatment (0.1, 1 or 10 μg/ml) induced significantly a drop in the mRNA expression of cytochrome *c* (11.57-fold, 5.20-fold, 1.71-fold respectively) and caspase-3 (8.69-fold, 4.69-fold, 2.43-fold respectively) compared with the control group in a dose-dependent manner.

## DISCUSSION

The present study showed that preincubation of H9c2 with catalpol (0.1–10 μg/ml) for 24 h markedly reduced the decrease in viability associated with exposure to H_2_O_2_ (100 μM) for 24 h as determined by MTT and LDH assay. MDA levels were significantly decreased and SOD activities were significantly increased by catalpol pretreatment in a dose-dependent fashion. Catalpol decreased H_2_O_2_-induced apoptosis in H9c2 cells in a concentration-dependent manner as indicated by Hoechst 33258 and flow-cytometric analysis. The mechanisms underlying the protective action of catalpol against H_2_O_2_-induced apoptosis appear to not only be associated with antioxidant, down-regulation of Bax and up-regulation of Bcl-2, but also involve the mitochondrial-dependent caspase pathway.

Catalpol, an iridoid glucoside, as the main active principle of *R. glutinosa* Libosch, possesses a wide variety of biological activities such as anti-tumour [9], anti-inflammation [10], anti-oxidation [19,20] and anti-apoptosis based on *in vitro* and *in vivo* pharmacodynamic experiments. Our previous works have demonstrated that catalpol could prevent HUVECs from death by inhibiting apoptosis and oxidative stress [11]. Most important is that in animal models, we found that catalpol could degrade apoptosis in transient global ischaemia in mouse and protect mice brain from oxidative damage and mitochondrial dysfunction induced by rotenone [21,22]. In the present study, we examined the effect of catalpol on the viability of H9c2 exposed to H_2_O_2_ by MTT and LDH assay. The H_2_O_2_-induced cell death was typical of apoptosis, both morphologically and biochemically, evidenced by Hoechst 33258 and Annexin V-binding. We found that catalpol protected H9c2 from H_2_O_2_-induced decreases in cell viability and apoptosis in a concentration-dependent manner.

Lipid peroxidation plays a pivotal role in tissue injury. To protect against oxidative damage, cells are often equipped with several antioxidants. Endogenous antioxidants, like SOD, can convert reactive oxygen species into less noxious compounds or prevent their formation, and it has been suggested to be protective against various forms of oxidative cardiovascular injuries [23]. MDA, a by-product of lipid peroxidation induced by free radicals, is widely used as a marker for oxidative stress [24]. In our study, the activities of SOD showed a statistically significant decline in H_2_O_2_ group compared with control group. Treatment with catalpol for 24 h could improve the activities of SOD in dose-dependent manner. In addition, an obvious enhancement of the level of MDA was shown in the H_2_O_2_ group, and it could be significantly reduced after catalpol administration. Therefore, our findings suggest that an increase in the activity of SOD and decrease in the concentration of MDA, consequently, decreased lipid peroxidative damage, which are involved in the mechanisms underlying the protective effect of catalpol against H_2_O_2_-induced injury.

Many genes have been reported to be linked with the regulation of programmed cell death under physiological and pathological conditions, among which, Bcl-2 acts to protect against apoptosis triggered by a wide range of factors and Bax plays as a death inducer within apoptotic pathways. Bcl-2 stabilizing mitochondrial membrane potential, and preventing the release of cytochrome *c* [25], also can form heterodimers with Bax and lose its protective effect. When Bcl-2 is present in excess, cells are protected from apoptosis. However, when Bax is in excess and the homodimers of Bax dominate, cells are susceptible to programmed cell death. The balance between pro-apoptotic and anti-apoptotic proteins plays a major role in determining the cell death or survival after apoptotic stimuli [26]. Obviously, the protective effects of catalpol against H_2_O_2_ toxicity observed here are, at least in part, attributed to its ability to suppress up-regulation of the pro-apoptotic protein Bax and elevate the expression of anti-apoptotic protein Bcl-2. Our previous studies were consist with this result that catalpol modulates the expressions of Bax and Bcl-2 and attenuates apoptosis in HUVECs after oxidant stress [11].

Mitochondria are common integrators and transducers of various pro-apoptotic signals, and its disruption and the subsequent release of pro-apoptotic proteins like cytochrome *c* is a critical event in the process of apoptosis in most systems [13,25,27]. Collapse of mitochondrial membrane potential (ΔΨ_m_) stated that an increase in mitochondrial cytochrome *c* is released into the cytosol. The released cytochrome *c* is regulated by Bcl-2 family proteins, antagonized by anti-apoptotic members and initiated by pro-apoptotic members. Cytochrome *c* plays a key role to form the apoptosoms by binding to procaspase-9 and apoptotic protease activating factor-1 (Apaf-1) in the presence of ATP [28]. The formation of the apoptosome then causes cleavage of procaspase-9 which is responsible for activating effector caspases, such as caspase-3, which is a key protease of the apoptotic machinery and causes cleavage of polyadenosine diphosphate ribose polymerase (PARP), and ultimately resulting in apoptosis [29]. We demonstrated that in H9c2 cells, exposure to 100 μM H_2_O_2_ for up to 24 h increased the release of cytochrome *c* and the activity of caspase-3. Catalpol markedly decreased the leaves of cytochrome *c* and the activity of caspase-3. These results suggest that catalpol may protect H9c2 from H_2_O_2_-induced apoptosis at least part by activating a mitochondrial-dependent caspase pathway.

In summary, our results clearly show that catalpol can attenuate H_2_O_2_-induced apoptosis in H9c2 *in vitro*. Our results also suggest that the underlining mechanisms of protective effects of catalpol are partly due to antioxidant, activation of the mitochondrial-dependent caspase pathway and altered Bcl-2 and Bax expression. These findings provide a new insight into the application for catalpol in the treatment of cardiovascular diseases.

## Abbreviations

DMEM: Dulbecco's modified Eagle's medium
HUVEC: human umbilical vein endothelial cell
LDH: lactate dehydrogenase
MDA: malondialdehyde
PI: propidium iodide
SOD: superoxide dismutase
